# Dermatological Manifestations of Amyloid Light-Chain (AL) Amyloidosis: A Case Report Highlighting Early Diagnosis and Treatment

**DOI:** 10.7759/cureus.99247

**Published:** 2025-12-15

**Authors:** Shubhkaran Singh Gill, Teenu Kamboj

**Affiliations:** 1 Department of Internal Medicine, Dayanand Medical College and Hospital, Ludhiana, IND

**Keywords:** amyloid deposits, amyloidosis al, clinical case report, congo red stain, early identification and diagnosis, immunoglobulin light-chain amyloidosis, monoclonal gammopathy, pinch purpura, primary cutaneous amyloidosis, secondary systemic amyloidosis

## Abstract

This case report describes a 52-year-old man who presented with gradually progressive dark cutaneous discoloration over the upper trunk and periorbital region, accompanied by painless enlargement of the tongue, which initially interfered with speech and eating. Over the subsequent months, he experienced marked unintentional weight loss, persistent fatigue, and symptoms consistent with autonomic involvement, including postural dizziness and altered bowel habits. Clinical examination revealed waxy skin changes and macroglossia.

Laboratory evaluation demonstrated a monoclonal light-chain abnormality, and additional testing identified abnormal serum protein bands and Bence-Jones protein in urine. Cross-sectional imaging showed hepatosplenomegaly without focal lesions, and biopsy of the abdominal fat pad confirmed amyloid deposition. Based on the constellation of clinical, laboratory, imaging, and histopathologic findings, a diagnosis of systemic amyloid light-chain (AL) amyloidosis was established.

The patient was initiated on bortezomib and dexamethasone chemotherapy targeting abnormal light-chain production, following which he exhibited progressive improvement in dermatologic changes, reduction in tongue size, improved energy levels, and stabilization of autonomic symptoms. This case underscores an atypical dermatologic onset of systemic AL amyloidosis, in which skin and tongue abnormalities preceded other systemic manifestations, enabling timely diagnosis and intervention.

## Introduction

Amyloidosis is a heterogeneous group of disorders characterized by the extracellular deposition of amyloid, an insoluble fibrillar protein, within tissues. These deposits disrupt normal tissue architecture and function, often leading to organ failure. Amyloid deposits can affect virtually every organ in the body, including the skin, heart, kidneys, liver, and nervous system [1]. Among the various forms of amyloidosis, primary amyloidosis--also known as light-chain amyloidosis (AL amyloidosis)--is caused by the deposition of immunoglobulin light chains produced by abnormal plasma cells. AL amyloidosis is the most common form of systemic amyloidosis and is typically associated with plasma cell dyscrasias such as multiple myeloma.

Dermatological manifestations are often a clue to the diagnosis of systemic amyloidosis, especially AL amyloidosis. Although amyloidosis is a multisystem disorder, cutaneous signs may be among the first indicators of the disease in 30 to 40% of patients with primary systemic amyloidosis [2]. Skin findings in amyloidosis are diverse and can range from asymptomatic purpura to more significant lesions such as plaques, nodules, and papules. Macular amyloidosis, lichen amyloidosis, and nodular amyloidosis are the three major cutaneous variants of localized amyloidosis; however, the cutaneous manifestations in systemic AL amyloidosis may mimic those of primary cutaneous amyloidosis but are usually associated with more severe systemic involvement.

Globally, AL amyloidosis is a relatively rare disease, with an estimated incidence of 8 to 12 cases per million person-years [3]. It predominantly affects older adults, with the median age at diagnosis being around 65 years, although cases have been reported in younger individuals [3]. The male-to-female ratio is approximately 1.5:1, reflecting a slight male predominance in disease occurrence [4]. Epidemiological studies indicate that the disease is more prevalent in Western countries and strongly correlates with the burden of underlying monoclonal gammopathies such as multiple myeloma [5].

In AL amyloidosis, the abnormal clonal plasma cells produce an excess of immunoglobulin light chains, which misfold and aggregate into amyloid fibrils. These fibrils are then deposited in tissues, including the skin. The skin, being a highly visible and accessible organ, often serves as a window to the diagnosis of this systemic condition. Common skin findings include purpura, particularly in areas of high venous pressure such as the eyelids, neck, and axillae. These purpuric lesions are caused by amyloid infiltration of blood vessel walls, leading to fragility and rupture, even with minimal trauma [6].

Another hallmark of cutaneous involvement in AL amyloidosis is macroglossia, or an enlarged tongue, which occurs due to amyloid deposition in the submucosa of the tongue. This condition is relatively specific to AL amyloidosis and is seen in approximately 10% to 20% of cases [7]. Macroglossia can cause difficulties with speech, swallowing, and breathing, and is often associated with dental indentations along the lateral edges of the tongue due to chronic pressure against the teeth [5,6]. Other mucocutaneous findings include petechiae, ecchymoses, and waxy papules, particularly in periorbital and flexural areas [8]. These waxy papules, which are often described as having a translucent, “pinched” appearance, can coalesce to form larger plaques.

Cutaneous manifestations in AL amyloidosis can mimic other dermatological conditions, making the diagnosis challenging. Therefore, clinicians need to maintain a high index of suspicion, especially in patients with known plasma cell disorders or those presenting with unexplained systemic symptoms such as fatigue, weight loss, or renal insufficiency [9]. Dermoscopic evaluation of cutaneous amyloid lesions has been shown to be a useful adjunct in the clinical diagnosis of amyloidosis. Dermoscopy can reveal characteristic findings, such as shiny white or yellowish dots and streaks in the papillary dermis, helping to distinguish amyloid deposits from other causes of pigmentation disorders [9].

Histopathological examination remains the gold standard for diagnosing amyloidosis. In AL amyloidosis, skin biopsy specimens typically show deposition of amorphous eosinophilic material in the dermis, which stains positively with Congo red and exhibits apple-green birefringence under polarized light. This characteristic staining pattern is crucial for confirming the presence of amyloid fibrils and differentiating amyloidosis from other conditions with similar clinical presentations [5,8]. Immunofixation electrophoresis and serology can further help differentiate AL amyloidosis from other forms of amyloidosis, such as amyloid A (AA) amyloidosis, by demonstrating the presence of kappa or lambda light chains [5,8].

The cutaneous findings in AL amyloidosis often parallel the severity of systemic involvement, particularly in the kidneys, heart, and nervous system. Therefore, the presence of dermatological signs should prompt a thorough workup to assess for systemic amyloid deposition. This typically includes serum and urine protein electrophoresis, immunofixation, and measurement of serum free light chains, which can help detect monoclonal light chains. Imaging studies, such as echocardiography and MRI, may be necessary to assess cardiac involvement, which is a major determinant of prognosis in AL amyloidosis [5]. Cardiac involvement is present in patients with AL amyloidosis and is associated with a poor prognosis due to restrictive cardiomyopathy and heart failure [5,7].

Despite advancements in treatment, AL amyloidosis remains a challenging disease to manage, with an overall median survival of approximately two to four years [10]. Early recognition and treatment are critical to improving outcomes, and dermatologists play a key role in identifying cutaneous manifestations of amyloidosis. The treatment of AL amyloidosis focuses on reducing the production of amyloidogenic light chains through chemotherapy and, in selected cases, autologous stem cell transplantation [5]. Emerging therapies, such as monoclonal antibodies targeting amyloid fibrils, are also showing promise in clinical trials [11].

## Case presentation

**Figure 1:**
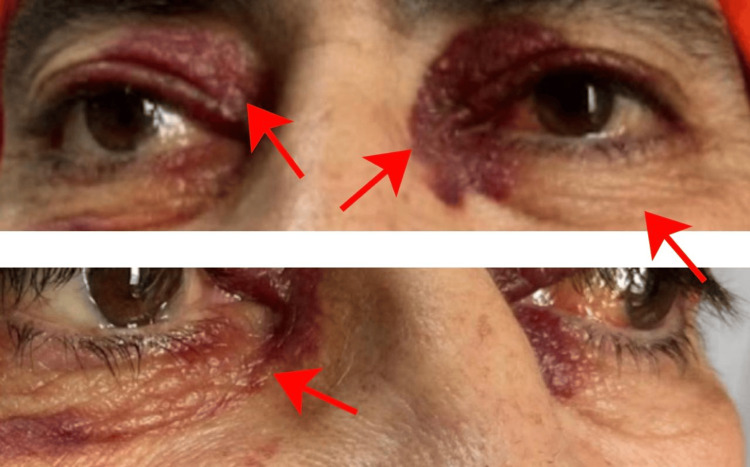
Patient before treatment showing periorbital lesions (as indicated by the arrows) Prominent bilateral periorbital purpura, ecchymosis patches, and periorbital edema are visible.

**Figure 2:**
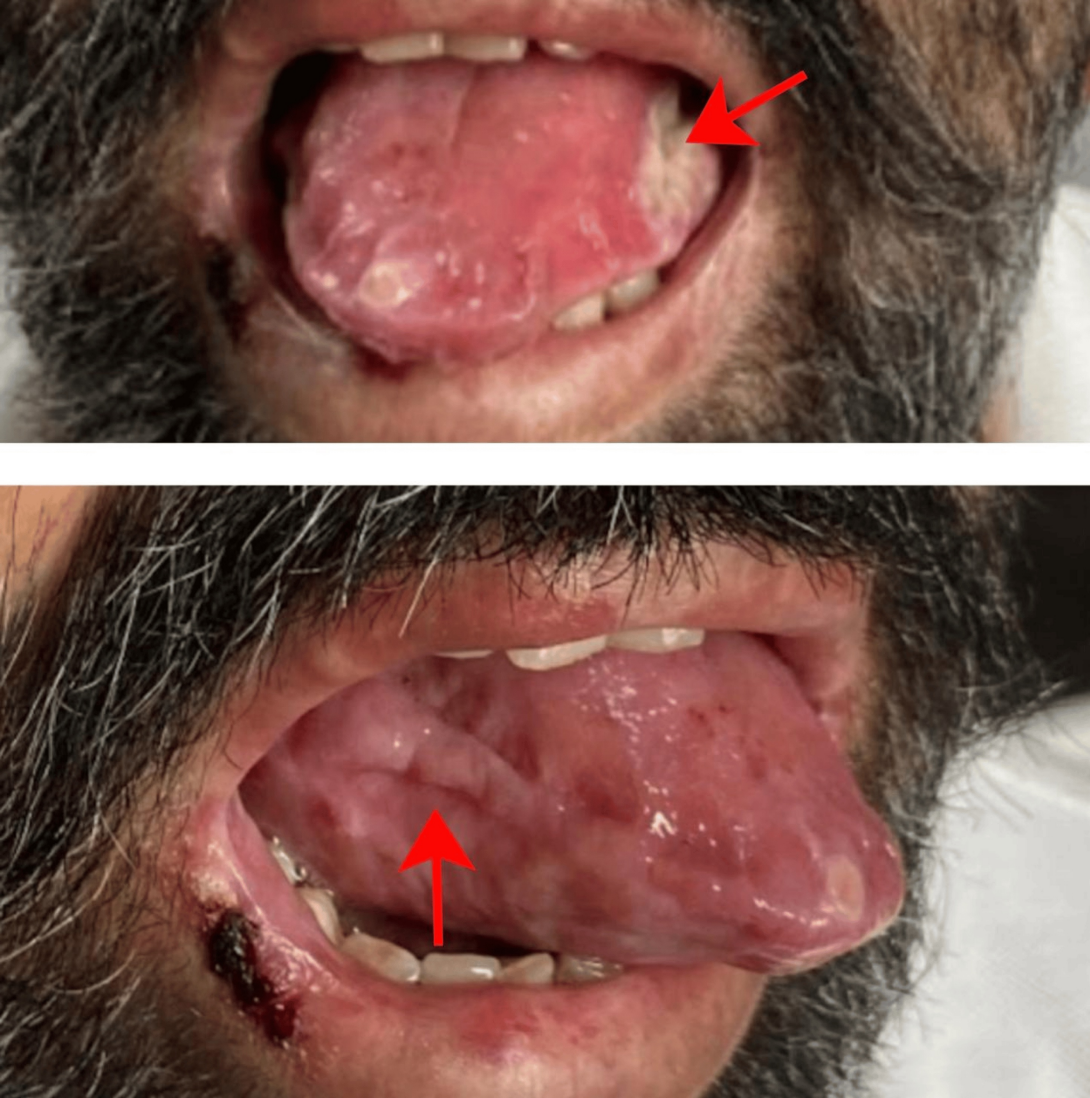
Patient before treatment showing macroglossia (as indicated by the arrows) Significant macroglossia with surface nodularity, reddish hue, and swelling. The lateral borders of the tongue are scalloped due to chronic pressure against the tongue.

A 52-year-old man with no significant medical history presented to the outpatient department with progressively worsening reddish, elevated pigmentation. The first signs appeared around the periorbital area (Figure 1) and gradually extended to his hands, upper back, and forearms, eventually affecting other parts of his body. About a week after the onset of skin lesions, he noticed changes in his tongue, including a reddish hue, swelling, and a heavy, bloated sensation, leading to macroglossia (Figure 2). 

This resulted in significant difficulties with eating and speaking, causing a dramatic weight loss of approximately 50 kg over 3 months. His inability to swallow and articulate properly exacerbated his malnutrition.

In addition to his dermatological and oral symptoms, the patient experienced profound fatigue, which worsened to the point where he became unable to walk or stand without assistance. He was bedridden for most of the day due to extreme weakness and lack of energy. The patient also developed new-onset orthostatic hypotension, causing dizziness and lightheadedness upon standing, further limiting his mobility.

**Figure 3:**
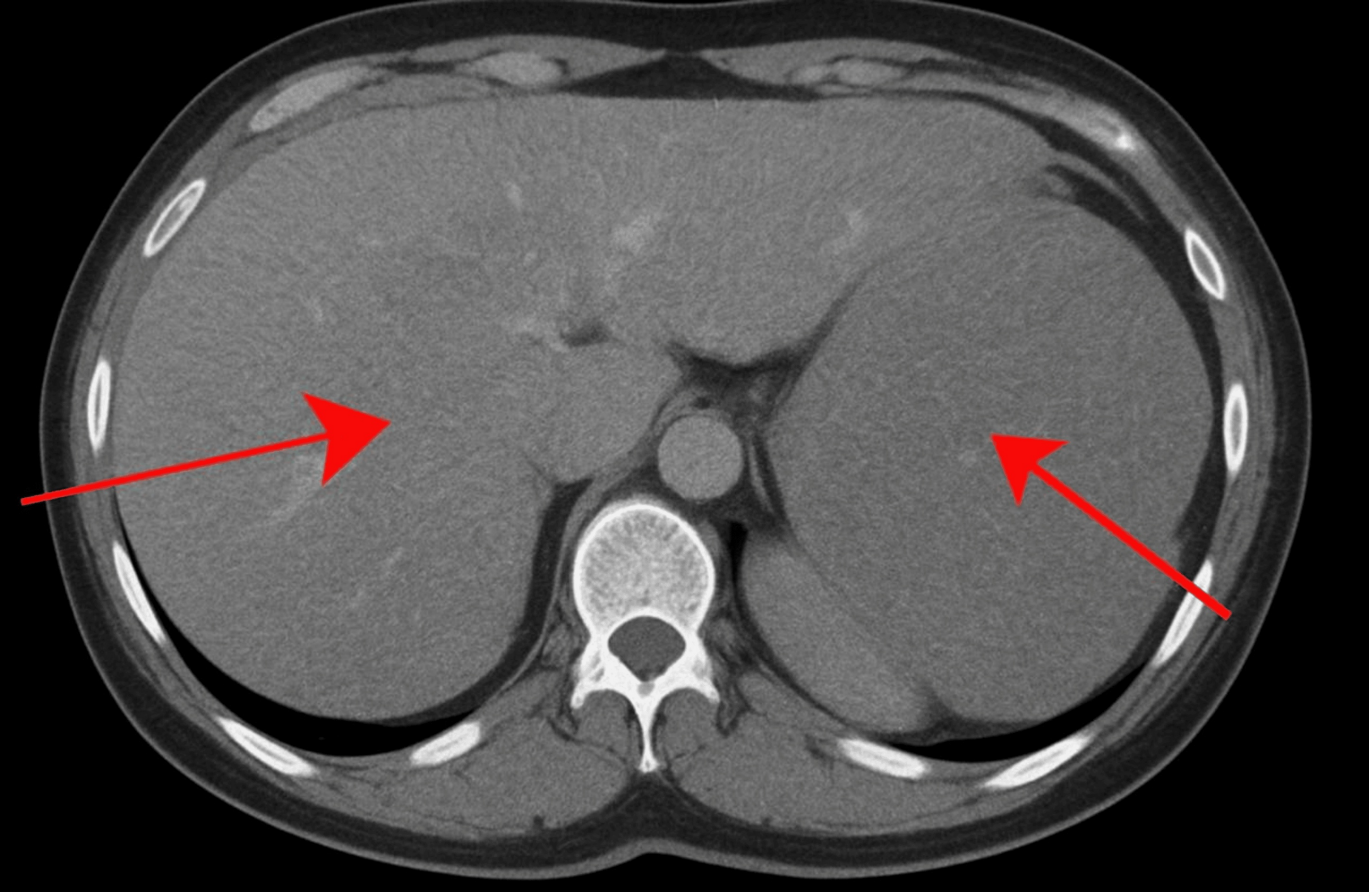
CT scan of the patient showing hepatosplenomegaly (as indicated by the arrows) Axial contrast-enhanced CT scan of the abdomen demonstrating hepatosplenomegaly in a patient presenting with dermatological manifestations of AL amyloidosis. Both liver and spleen are diffusely enlarged with no focal hepatic or splenic lesions, reflecting systemic amyloid deposition.

Upon admission to the hospital, the patient underwent a comprehensive diagnostic workup. The complete details of this diagnostic assessment are summarized in Table 1. The key radiological findings are illustrated in Figure 3.

**Table 1:** Diagnostic workup of the patient

Category	Test Parameter	Result	Reference Range	Units / Notes
Hematology	Hemoglobin	9.4 – Mild normochromic, normocytic anemia	13.5–17.5 (M) / 12.0–15.5 (F)	g/dL
	Mean Corpuscular Volume (MCV)	Within normal range	80–100	fL
	Mean Corpuscular Hemoglobin Concentration (MCHC)	Within normal range	32–36	g/dL
	White Blood Cell Count	Within normal limits	4.0–11.0	×10⁹/L
	Platelet Count	Within normal limits	150–450	×10⁹/L
Protein Studies	Serum Protein Electrophoresis (SPEP)	Distinct monoclonal spike (1.92 g/dL)	No M-spike detectable	Qualitative
	Urine Protein Electrophoresis (UPEP)	Positive for Bence-Jones proteins	Should be absent	Qualitative
Light Chain Analysis	Serum Free κ Light Chains	4.1 – Reduced relative to λ	3.3–19.4	mg/L
	Serum Free λ Light Chains	145.2 – Markedly elevated	5.7–26.3	mg/L
	κ/λ Ratio	0.028 – Significantly decreased	0.26–1.65	Ratio
Imaging	Whole-Body CT Scan	Hepatosplenomegaly; no focal solid lesions (as illustrated in figure 2)	—	Indicative of systemic involvement
Histopathology	Abdominal Fat Pad Biopsy	Congo red-positive deposits with apple-green birefringence	Should be negative	Congo red staining under polarized light
	Birefringence Evaluation	Apple-green birefringence observed	Should be absent	Polarized light microscopy

The patient was initiated on a treatment regimen aimed at reducing the production of amyloidogenic light chains by targeting the underlying plasma cell disorder. The regimen consisted of weekly injections of bortezomib (dose 2.3 mg, based on 1.3 mg/m²), a proteasome inhibitor, in combination with dexamethasone. This combination was selected due to its proven efficacy in reducing monoclonal light chains and improving organ function.

**Figure 4:**
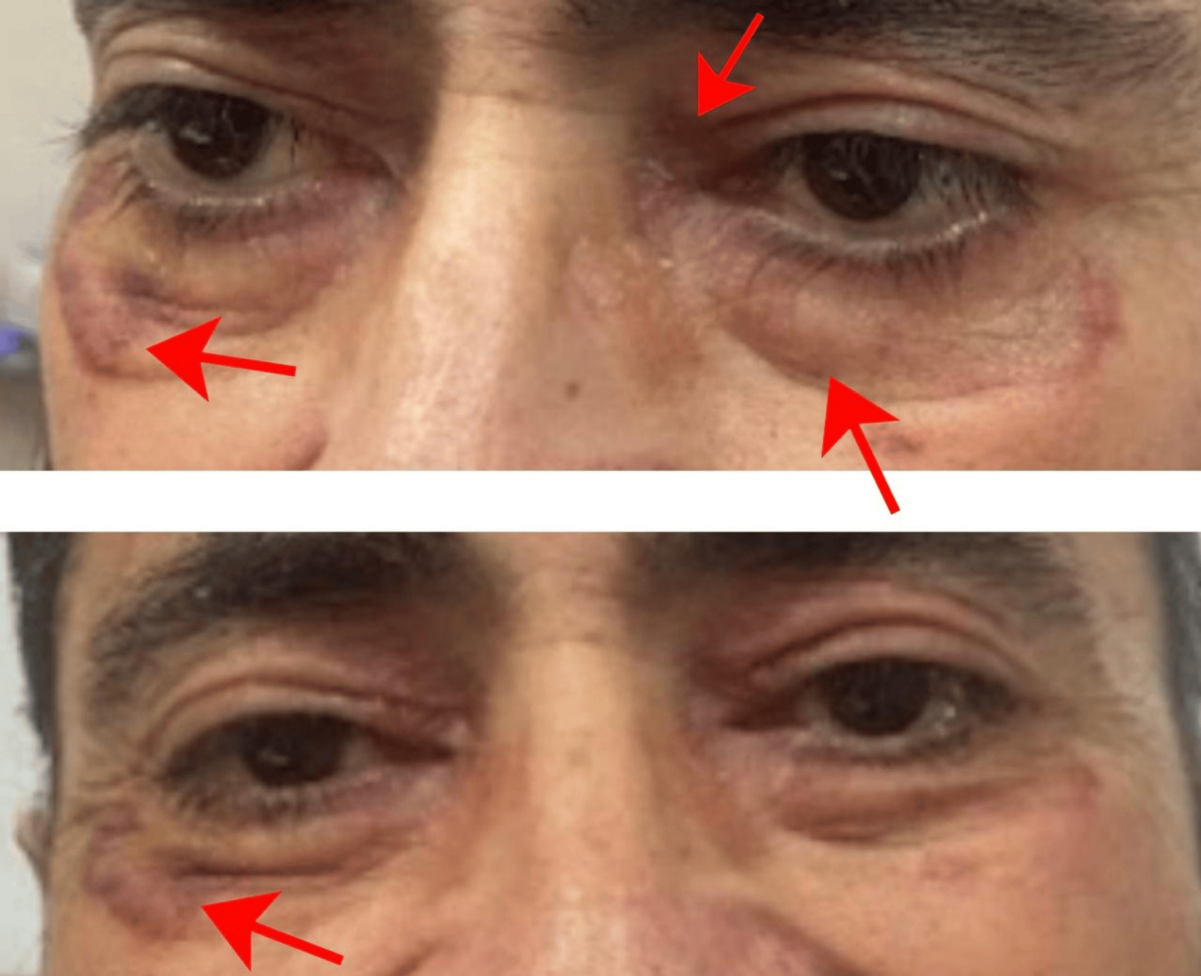
Patient after treatment with bortezomib and dexamethasone for four weeks showed improvement in periorbital edema and lesions around the eyes (as indicated by the arrows) Resolution of amyloid-related soft-tissue manifestations after four weeks of treatment with bortezomib and dexamethasone. Ecchymosis patches improved, bilateral periorbital purpura and edema showed significant improvement.

**Figure 5:**
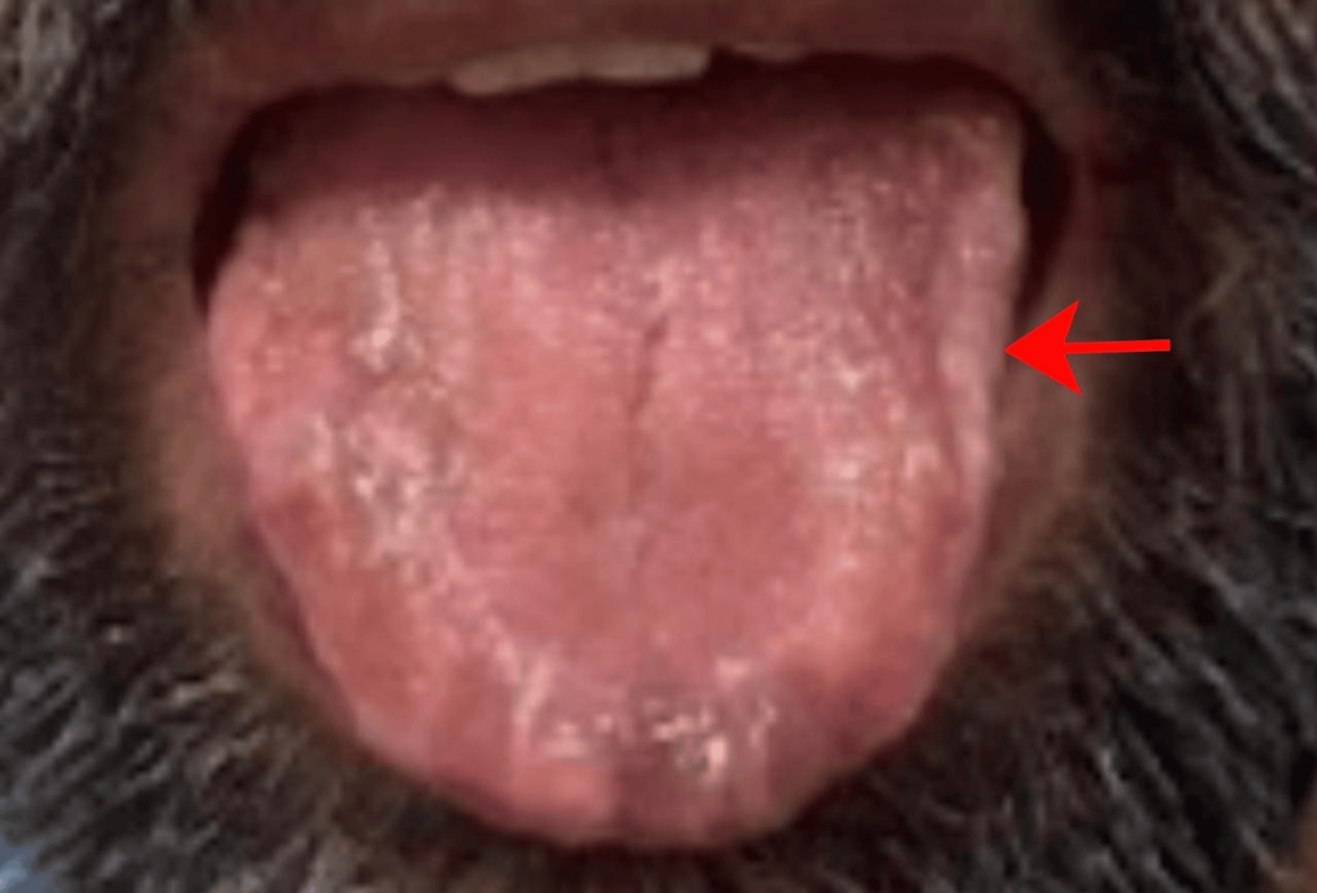
Patient after treatment with bortezomib and dexamethasone for four weeks showed a reduction in macroglossia (as indicated by the arrows) There was a significant reduction in macroglossia, and the scalloped tongue borders markedly improved.

After four weeks of treatment, the patient showed significant improvement. The pigmentation lesions became less prominent (Figure 4), and the swelling of his tongue subsided (Figure 5). He was able to eat comfortably, and his energy levels increased, enabling him to walk with assistance.

The patient completed eight doses of bortezomib and was transitioned to a maintenance regimen with subcutaneous bortezomib (dose: 2.3 mg subcutaneously every 2 weeks). By March 2024, he experienced a remarkable recovery, regaining his ability to walk and engage in daily activities, including driving.

The patient is being closely monitored through regular follow-ups. While his skin lesions have improved, they tend to recur with minimal trauma, indicating the need for continued dermatological care. His cardiac and renal functions have remained stable, with no further evidence of amyloid deposition on follow-up imaging.

Given the chronic and systemic nature of AL amyloidosis, the patient’s prognosis remains guarded. However, his positive response to treatment is encouraging, and he continues to receive maintenance therapy to prevent relapse. Ongoing monitoring of light chain levels, organ function, and skin lesions will be essential to manage the disease and prevent complications.

## Discussion

AL amyloidosis is a rare systemic disease resulting from the deposition of misfolded immunoglobulin light chains in various tissues, leading to significant morbidity and mortality. Dermatological manifestations, although less commonly the initial presentation, are a key diagnostic clue in AL amyloidosis and may offer early insight into the underlying systemic pathology. The skin, due to its accessibility, serves as an important diagnostic window to recognize the systemic involvement of amyloid deposition. A similar case is of a 26-year-old male who presented with closely set skin-coloured papules on the eyelids, around the nose and mouth, and over the scalp, and a history of generalized weakness, hoarseness of voice, and an increase in the size of his tongue. Histopathology showed clumps of eosinophilic material, which were positive for the Congo red stain. There was also proteinuria, and a final diagnosis of myeloma-associated systemic amyloidosis was made. The patient started bortezomib-cyclophosphamide-dexamethasone treatment cycles every 28 days. In this case, the dermoscopic examination helped detect early vascular changes, prior to the clinical appearance of classically evident papules of systemic amyloidosis. A high degree of suspicion and early dermoscopic evaluation can help prompt a systemic amyloidosis diagnosis with cutaneous involvement [9]. In this discussion, we explore both common and uncommon cutaneous manifestations of AL amyloidosis, evaluate treatment options, discuss the natural course and prognosis of the disease, and outline potential complications that may arise.

Common dermatological manifestations

Purpura is one of the hallmark dermatological signs of AL amyloidosis. These lesions are often seen on the face, especially around the eyelids, neck, and upper chest. This "pinch purpura" is a common finding, usually exacerbated by minor trauma due to amyloid deposition in blood vessel walls, leading to vascular fragility. Purpuric lesions may also appear on flexural areas of the body, and in severe cases, widespread ecchymoses may develop. These lesions result from amyloid’s tendency to infiltrate dermal blood vessels, causing vascular fragility, which contributes to their pathognomonic presentation [6]. Additionally, periorbital purpura, which manifests as a characteristic raccoon-eye appearance, can be a striking early manifestation, providing a visual clue for systemic amyloidosis, especially in the absence of significant trauma [12].

Another common cutaneous feature in AL amyloidosis is waxy, translucent papules and plaques that tend to localize in flexural areas such as the neck, axillae, and groin. These papules, often described as having a "waxy" or "pinched" appearance, may coalesce to form plaques, giving the skin a wax-like consistency. Histopathologically, these lesions exhibit characteristic amyloid deposits in the dermis, which stain positively with Congo red, producing apple-green birefringence under polarized light [5,8]. The waxy plaques tend to have an insidious onset and are often asymptomatic, though they may become more pronounced over time.

Macroglossia, or tongue enlargement, is a relatively specific manifestation of AL amyloidosis and occurs in approximately 15% of cases [5]. This condition arises from the deposition of amyloid in the submucosa of the tongue, causing significant enlargement, which may interfere with speech, mastication, and swallowing. Patients often present with difficulties in articulation, dysphagia, and airway obstruction in severe cases. Characteristic dental indentations along the lateral borders of the tongue may be seen due to chronic pressure against the teeth [6]. Macroglossia is a diagnostic red flag for AL amyloidosis, as it is rarely seen in other forms and is highly suggestive of systemic amyloidosis, and it should prompt further investigation into systemic involvement [5].

Uncommon dermatological manifestations

While purpura and waxy papules are the most frequently observed dermatological features of AL amyloidosis, other rare skin manifestations can also occur, though they are less commonly recognized. Nodular amyloidosis is one such variant, where amyloid deposits manifest as firm, skin-colored, or slightly hyperpigmented nodules. These nodules may mimic benign or malignant neoplasms, making clinical diagnosis challenging without histological confirmation. Nodular amyloidosis is rare but can indicate the presence of systemic involvement. The clinical appearance of nodules can be mistaken for granulomatous conditions or even squamous cell carcinoma, highlighting the need for a biopsy to confirm the diagnosis [13].

Macular amyloidosis, although more common in primary localized cutaneous amyloidosis, can also appear in AL amyloidosis. It presents as hyperpigmented macules, often with a rippled or reticulated pattern, particularly on the extensor surfaces of the upper back or arms. This presentation is less commonly associated with systemic disease but should still be considered as part of the amyloidosis spectrum, especially when coexisting with other systemic symptoms.

Poikiloderma-like changes, including skin atrophy, telangiectasias, and pigmentation abnormalities, have also been reported in a subset of patients with AL amyloidosis. These findings can mimic other dermatological conditions, such as dermatomyositis or cutaneous T-cell lymphoma, making clinical differentiation challenging. The presence of such findings should prompt dermatologists to maintain a high index of suspicion, particularly in patients with systemic symptoms such as fatigue, weight loss, or organ dysfunction [12].

Treatment of dermatological manifestations in AL amyloidosis

The primary treatment of AL amyloidosis revolves around reducing the production of amyloidogenic light chains by targeting the underlying plasma cell disorder, which is crucial for preventing further organ damage [5,14]. Chemotherapy regimens, often incorporating proteasome inhibitors, such as bortezomib combined with dexamethasone, have shown efficacy in reducing light-chain production and improving overall survival in patients with AL amyloidosis [5,14]. A striking difference has been observed with the introduction of bortezomib, which results in clonal response rates of 70-90%, including around 40% of CR [5]. Bortezomib-based regimens have become the cornerstone of therapy in AL amyloidosis due to their rapid and deep hematologic response rates. Clinical studies report hematologic response rates of 60-80%. Organ responses, particularly cardiac and renal, typically follow hematologic improvement and occur in 30-40% of cases. Bortezomib has also been used successfully in patients with multiple myeloma and renal dysfunction [14]. The addition of dexamethasone enhances the depth and speed of response, making the bortezomib-dexamethasone combination a widely used first-line regimen in systemic AL amyloidosis. There is a significant in vitro and in vivo synergism between bortezomib and dexamethasone, both in pretreated and in newly diagnosed patients with multiple myeloma [14]. These regimens not only help in controlling systemic involvement but also improve skin-related symptoms by reducing amyloid deposition over time. Other treatments may include daratumumab, which is a monoclonal antibody targeting CD38 and used for multiple myeloma treatment [14]. Patients often report improvement in cutaneous manifestations such as waxy papules and purpura within a few months of starting therapy.

In addition to systemic treatment, topical and surgical interventions may be considered for localized skin lesions. For example, topical retinoids have been used to treat pruritic macular amyloidosis, with some success in reducing pigmentation and pruritus. However, in cases of more severe cutaneous involvement, such as extensive nodules or large plaques, surgical excision may be necessary to alleviate discomfort or prevent further complications [15]. Cryotherapy and laser ablation have also been used as adjunctive treatments for amyloid deposits in the skin, though their role remains limited to case reports [16].

Natural course and prognosis

The natural course of AL amyloidosis is progressive and depends heavily on the extent of systemic involvement, particularly in the kidneys and heart. Dermatological manifestations may evolve over time, with the initial presentation of purpura or waxy papules potentially worsening if left untreated. In some cases, cutaneous signs may persist or recur despite systemic treatment, indicating ongoing amyloid deposition. However, skin manifestations generally respond well to systemic therapies that control the underlying plasma cell dyscrasia, and their improvement often parallels the resolution of systemic symptoms [17].

The prognosis of patients with AL amyloidosis remains guarded, primarily due to the frequent involvement of vital organs, but survival is improving [10]. Cardiac involvement, present in approximately 50% of cases, is a major determinant of prognosis, with patients who have significant cardiac amyloidosis exhibiting markedly reduced survival [5,7]. The cardiac evaluation of the patient by echocardiogram was not done in this case report due to the emergent nature of the patient's presentation and resource limitations, which is a limitation of the diagnostic evaluation. Early identification and treatment are crucial for improving outcomes, and the presence of dermatological signs should prompt a thorough evaluation for systemic disease. 

Patients with AL amyloidosis who receive prompt treatment with modern chemotherapy regimens have a median survival of approximately two to four years, though outcomes vary widely depending on the severity of organ involvement. Autologous stem cell transplantation may offer long-term remission in selected patients, though it is not suitable for all due to the potential for treatment-related complications [18].

Complications

Complications in AL amyloidosis often stem from systemic involvement, but cutaneous manifestations can also lead to significant morbidity. For example, macroglossia may cause airway obstruction, dysphagia, particularly in patients with advanced disease [5,7]. Patients with severe macroglossia may require surgical intervention or speech therapy to improve their quality of life. Cutaneous purpura and ecchymoses, although typically asymptomatic, can cause cosmetic concerns and increase the risk of secondary infection, especially if trauma occurs in areas with extensive amyloid deposition [19,20].

Nodular amyloidosis, while uncommon, can lead to ulceration or secondary infection, particularly if lesions are located in areas subject to frequent friction or trauma. In rare cases, nodular amyloidosis may undergo malignant transformation into squamous cell carcinoma, though this is exceedingly rare and primarily reported in isolated case studies [20]. Other complications may include pruritus, particularly in patients with macular amyloidosis, which can be severe and refractory to standard treatments [21].

Dermatologists play a pivotal role in the long-term management of patients with AL amyloidosis, as cutaneous signs can serve as markers for disease recurrence or progression. Routine skin examinations and biopsies may be necessary in patients with known systemic amyloidosis, particularly if new lesions develop or if existing lesions change in appearance.

## Conclusions

The cutaneous manifestations of AL amyloidosis offer critical diagnostic clues and should not be overlooked in the evaluation of patients with systemic symptoms. Dermatological features, such as purpura, waxy papules, and macroglossia, are often the first signs of disease and can guide clinicians toward a timely diagnosis of systemic amyloidosis. While the treatment of AL amyloidosis focuses on reducing amyloidogenic light chain production, dermatological management also plays an important role in alleviating symptoms and improving patient outcomes. Early recognition, combined with a multidisciplinary approach, is essential for optimizing the prognosis in patients with this challenging multisystem disease.
